# Relation between sleep quality and quantity, quality of life, and risk of developing diabetes in healthy workers in Japan: the High-risk and Population Strategy for Occupational Health Promotion (HIPOP-OHP) Study

**DOI:** 10.1186/1471-2458-7-129

**Published:** 2007-06-28

**Authors:** Yasuaki Hayashino, Shunichi Fukuhara, Yoshimi Suzukamo, Tomonori Okamura, Taichiro Tanaka, Hirotsugu Ueshima, the HIPOP-OHP Research group

**Affiliations:** 1 Department of Epidemiology and Healthcare Research, Kyoto University Graduate School of Medicine, Kyoto, Japan; 2 Department of Physical Medicine and Rehabilitation, Tohoku University Graduate School of Medicine, Sendai, Japan; 3 Department of Health Science, Shiga University of Medical Science, Shiga, Japan; 4 Members of the HIPOP-OHP Research group members are listed in the appendix, Japan

## Abstract

**Background:**

The effect of sleep on the risk of developing diabetes has not been explored in an Asian population. The objective of this study is to investigate the effect of self-reported sleep duration and sleep quality on the risk of developing diabetes in a prospective cohort in Japan.

**Methods:**

Data were analyzed from the cohort of participants in a High-risk and Population Strategy for Occupational Health Promotion Study (HIPOP-OHP), conducted in Japan from the year 1999 until 2004. A Cox proportional hazard model was used to evaluate the association between sleep duration or sleep quality and the risk of diabetes.

**Results:**

Of 6509 participants (26.1% of women, 19–69 years of age), a total of 230 type 2 diabetes cases were reported over a median 4.2 years of follow-up. For participants who often experienced difficulty in initiating sleep, the multivariate-adjusted hazard ratios for diabetes were 1.42 (95%CI, 1.05–1.91) in participants with a medium frequency of difficulty initiating sleep, and 1.61 (95%CI, 1.00–2.58) for those with a high frequency, with a statistically significant linear trend. Significant association was not observed in the association between difficulty of maintaining sleep or duration of sleep, and risk of diabetes.

**Conclusion:**

Medium and high frequencies of difficulty initiating sleep, but not difficulty in maintaining sleep or in sleep duration, are associated with higher risks of diabetes in relatively healthy Asian workers, even after adjusting for a large number of possible further factors.

## Background

Sleep quantity and quality has been reported to be associated with morbidity and mortality [1-4]. Experimental restriction of sleep to less than 4 hours per night for 6 nights resulted in impaired glucose tolerance in young healthy adults [5]. Since diabetes mellitus carries a high risk of cardiovascular mortality, the impact of sleep restriction on glucose regulation suggests a mechanism whereby short sleep time could increase mortality. Evidence is accumulating concerning the long-term effect of abnormal quality of sleep (e.g. difficulty initiating and maintaining sleep) or quantity of sleep (sleep duration) on the future risk of developing diabetes. Most of these studies were in the USA and Europe [6-8], and it is not clear whether the results are applicable to the Asian population. Evidence increasingly suggests that the epidemiology of type 2 diabetes depends on ethnicity [9].

The present study aims to investigate the relation between difficulty initiating or maintaining sleep, based on self-reported typical sleep duration, and quality of life; and to determine the effect of quality or quantity of sleep on the risk of developing diabetes in a large sample of healthy workers in Japan.

## Methods

### Participants and follow-up

We obtained the baseline and annual follow-up data during 1999–2004 from the High-risk and Population Strategy for Occupational Health Promotion Study (HIPOP-OHP). This study has been comprehensively described elsewhere [10-12]. In summary, this 4-year worksite trial began in Japan in 1999 for the purpose of preventing cardiovascular disease. Health promotion consisted of two approaches: health promotion intervention, and minimization of high-risk conditions associated with cardiovascular diseases. The intervention areas included diet, physical activity, and cessation of smoking. A total of 7226 participants were assigned to twelve worksites, with an intervention group and a control group of six worksites each. We excluded 717 participants from the analysis who had prevalent diabetes at the baseline survey conducted between 1999 and 2000, or those who did not report the quality or quantity of sleep (difficulty initiating sleep, difficulty maintaining sleep, and duration of sleep) at baseline. We therefore used a cohort of 6509 healthy male and female workers aged 19–69.

The intervention plan was carried out following ethics approval by the Safety Hygiene Committee of each company. The Ethics Committee of Shiga University of Medical Science consented to the research protocol of the present study (No. 10–16). Written informed consent was obtained from all participants receiving individual guidance under the high-risk strategy.

### Study variables and standardization

In 1999 or 2000, a baseline health check was performed on all participants. Data from the resulting physical examinations, including blood pressure, height, weight, and biological data such as plasma glucose level and cholesterol, were measured at baseline, and thereafter at annual check-ups. Details of whether participants were in a fasting state were also collected. History of diabetes and other lifestyle parameters, such as daily alcohol intake, smoking habits, use of medications, and exercise, were evaluated using a self-administered questionnaire [10-12]. BMI was calculated as weight (kilograms) divided by height (meters) squared. For recreational physical activities, a metabolic equivalent task (MET) score was assigned based on the energy cost of each activity [13], and the equivalent energy expenditure in hours per week (MET-h/w) for each participant was then estimated. Drinking habits were also assessed by a self-administered questionnaire; the frequency of alcohol consumption during a typical week and the total alcohol intake on each occasion was determined and used to calculate the alcohol intake per day.

Sleep duration and difficulty initiating sleep was evaluated at baseline using a self-administered questionnaire. Participants were asked the following questions: "Indicate the average hours of sleep you usually take on weekdays", "How often do you experience difficulty initiating sleep? 1. Often, 2. Sometimes, 3. Never", and "How often do you experience difficulty maintaining sleep? 1. Often, 2. Sometimes, 3. Never". Because the duration of sleep was not normally distributed, this variable was divided into four categories for the purpose of this analysis: < 6 hours; >= 6 hours and < 7 hours; >= 7 hours and < 8 hours; >= 8 hours.

To assess whether the questions regarding difficulty in initiating sleep and difficulty maintaining sleep were accurately assessing the target qualities regarding sleep, we evaluated the construct validity of this question. Construct validity refers to the extent to which an instrument is consistent with the hypothesis derived theoretically from the construct of interest. It has been reported that insomnia is associated with a low quality of life [14], and we therefore hypothesized that people with difficulty initiating sleep or difficulty maintaining sleep have a low quality of life. We therefore evaluated the association between difficulty initiating sleep and quality of life. Quality of life was evaluated using the Japanese version of the Short Form 36 health survey (SF-36) [15]. The SF-36 is a 36 item questionnaire which measures health using eight scales; it is a common measure of quality of life in studies of patients and the general population [16-21]. Cross-sectional data from population studies have shown that the SF-36 is reliable and can detect differences between groups defined by age, sex, socioeconomic status, geographical region, and clinical conditions [15]. The SF-36 is therefore be a useful tool for evaluating the difference between differing categories of difficulty in initiating sleep. For the present HIPOP-OHP study, only data from 22 items out of the 36 and from 5 of the sub-domains (vitality, mental health, role limitations-physical, role limitations-emotional, and general health) were collected. From these, we derived summary scores (0–100) for these 5 sub-domains in order to evaluate the association with the different categories of difficulty initiating sleep. For the purpose of validating the questions, we also hypothesized that participants experienced a shorter sleep duration if they had poor sleep quality, so that we evaluated the association between difficulty initiating sleep, difficulty maintaining sleep, and sleep duration.

### Diagnosis of diabetes

In accordance with the American Diabetes Association and World Health Organization, a case of diabetes was considered confirmed if the following criteria were met: 1) a fasting blood glucose level of 126 mg/dL or greater (>= 7.0 mmol/L), 2) a random plasma glucose of at least 200 mg/dl (11.1 mmol/L), 3) treatment with hypoglycemic medication (insulin or oral hypoglycemic agent). Self-reported history of diabetes was also taken to constitute a case, because it is reportedly reliable [22], and it has often been used as a measure of exposure or outcome in many cohort studies [23,24].

### Statistical analyses

We used direct standardization to compare categorical variables adjusted for age, and used the generalized linear model to compare age-adjusted continuous measurements. Because the alcohol intake variable had 8.9% of missing values, we modeled dummy variables for missing values of this variable. To determine the validity of the question concerning difficulty initiating sleep or difficulty maintaining sleep, we used the general linear model, and tested the trend of the quality of life scores across different categories of difficulty initiating sleep. To analyze the association between difficulty initiating sleep, difficulty maintaining sleep, and sleep duration, and incident diabetes cases, we used the Cox-proportional hazard model. Person-time was calculated from the return of the baseline questionnaire until the date of onset of diabetes or the end of follow-up, whichever occurred first. The association between difficulty initiating sleep and difficulty maintaining sleep categories and sleep duration categories was determined using the chi-square test. The generalized linear model was used to test the linear trend between the SF-36 sub-domain scores and the difficulty initiating sleep or the difficulty maintaining sleep.

We evaluated the effect of difficulty initiating sleep, difficulty maintaining sleep, and sleep duration on the risk of developing diabetes in separate age-adjusted and multivariable-adjusted models. For evaluation of sleep quality, the first multivariable model (model 1) controlled for variables that might confound the association between the relevant categories and incident diabetes cases. This model controlled for age (in 5-year increments), gender, physical activity (MET-h/w) quartiles, body mass index quartiles, history of smoking (never, past, current), history of hypertension, history of high cholesterol, parental history of diabetes, alcohol use (mg/week) quartiles, and assigned intervention (health promotion) of the original study. The second multivariable model (model 2) controlled for the duration of sleep categories, for the purpose of evaluating the association between difficulty initiating or maintaining sleep and the risk of developing diabetes. In the evaluation of sleep duration, we used slightly different models; BMI was not a factor in model 2 but was a factor in model 3, and the sleep duration categories were not included as an independent variable in any of the models for the nature of the analysis. We calculated age- and multivariable-adjusted hazard rates as a measure for the hazard ratio (HR) and the corresponding 95% confidence intervals (CIs).

We finally evaluated the joint association between sleep quality or sleep quantity and gender if it was associated with risk of diabetes, because recent studies reported gender differences in sleep disorders [7]. Likelihood ratio tests were used to test statistical interactions between sleep and gender by comparing the -2 log(likelihood) value between two nested models; one model had only the main effects, the other included the main effects and interaction terms. All analyses were performed using commercially available statistical software packages (Intercooled STATA 8.2, Stata Corp., College Station, Tex).

## Results

Of the 6509 participants included in the current analysis, the average age (range) and body-mass index at baseline were 38.2 (19–69) years and 22.6 kg/m^2^, suggesting that the study population consisted of relatively young and lean workers. The baseline frequencies of participants' difficulty initiating sleep are shown in Table 1. At baseline, 61.3% of participants reported no difficulty initiating sleep, 30.7% reported sometimes, and 8.0% reported often. At baseline, a high frequency of difficulty initiating sleep was associated with a higher occurrence of diabetes in family history, and a higher occurrence of a history of hypertension. Also, participants who reported often experiencing difficulty initiating sleep consumed more alcohol and exercised less, but were less likely to be current smokers.

Over a median 4.2 years of follow-up, a total of 230 diabetes cases were reported. After adjusting for age, the risk of developing diabetes proved similar in the various sleep duration categories (<6, 6–7, 7–8, >= 8) (Table 2). These associations remained unchanged even after adjusting for the large number of possible confounding factors.

**Table 1:** Baseline characteristics of the study participants according to difficulty initiating sleep categories, 1999–2004, HIPOP-OHP, Japan

	**Difficulty initiating sleeping**		
	**Non**	**Sometimes**	**Often**
No. of subjects (%)	3993 (61.3)	1996 (30.7)	520 (8.0)
Age *	38.4 ± 9.5	38.3 ± 9.5	36.2 ± 9.5
Gender (male, %)	77.7	78.6	83.0
BMI (kg/m^2^) † ‡	22.7 ± 0.05	22.7 ± 0.07	22.8 ± 0.14
Physical activity (MET-h/week) † §	5.5 ± 0.17	4.3 ± 0.24	5.1 ± 0.46
History of hypercholesterolemia (%)	7.5	7.8	8.4
History of hypertension (%)	11.8	13.1	13.6
Smoking (%)			
Never	44.4	37.1	30.0
Past	15.9	12.6	14.1
Current	39.7	50.3	55.9
Family history of diabetes (%)	18.2	19.1	17.8
Sleeping time (hours) (%)			
<6	14.3	16.9	30.3
6–7	41.9	44.6	42.4
7–8	34.3	31.6	21.9
8<	9.5	6.9	5.4
Alcohol intake quartiles (%)			
1Q (0 mg/week)	44.2	44.6	46.0
2Q (0.1–2.5 mg/week)	1.4	1.2	1.0
3Q (2.6–25.0 mg/week)	23.8	23.1	19.0
4Q (25.1-mg/week)	22.2	21.0	24.5
Data missing	8.4	10.1	9.5

* Plus-minus values are means ± SD

† Plus-minus values are means ± SE

‡ BMI denotes body mass index

§ MET-h denotes metabolic equivalent hours

**Table 2:** Self-reported sleep duration, difficulty initiating sleeping, difficulty maintaining sleeping and risk of type 2 diabetes, 1999–2004, HIPOP-OHP, Japan

**Self-reported sleep duration**	Age-adjusted Model*	**Model 1 †**	**Model 2 ‡**
< 6 hours	1.19 (0.81–1.76)	1.15 (0.76–1.74)	1.15 (0.76–1.74)
6–7 hours	1.00	1.00	1.00
7–8 hours	1.24 (0.92–1.69)	1.15 (0.84–1.59)	1.15 (0.84–1.59)
> 8 hours	1.16 (0.72–1.87)	1.02 (0.62–1.70)	1.03 (0.62–1.70)
P for trend	0.549	0.933	0.723

**Self-reported difficulty initiating sleeping**	Age-adjusted Model*	**Model 1 §**	**Model 2 ¶**

None	1.00	1.00	1.00
Sometimes	1.33 (1.00–1.76)	1.39 (1.04–1.88)	1.42 (1.05–1.91)
Often	1.65 (1.07–2.54)	1.63 (1.04–2.59)	1.61 (1.00–2.58)
P for trend	0.007	0.011	0.005

**Self-reported difficulty maintaining sleeping**	Age-adjusted Model*	**Model 1 §**	**Model 2 ¶**

None	1.00	1.00	1.00
Sometimes	1.31 (0.99–1.73)	1.31 (0.97–1.77)	1.31 (0.97–1.76)
Often	1.38 (0.91–2.11)	1.34 (0.86–2.01)	1.37 (0.87–2.16)
P for trend	0.043	0.074	0.063

* Adjusted for age at baseline (5 year increments)

† Adjusted for age, gender, history of smoking, history of hypertension, history of high cholesterol, potential history of diabetes, exercise (MET-h/week) quartiles, and assigned intervention (health promotion)

‡ Adjusted for all variables in model 1 and BMI (kg/m^2^, quartiles)

§ Adjusted for age, gender, BMI (kg/m^2^, quartiles), history of smoking, history of hypertension, history of high cholesterol, potential history of diabetes, exercise (MET-h/week) quartiles, and assigned intervention (health promotion)

¶ Adjusted for all variables in model 1 and sleep duration categories

A higher frequency of difficulty initiating sleep was associated with a significantly increased risk of developing incident diabetes in the age-adjusted model (Table 2). For participants who often experience difficulty initiating sleep, the age-adjusted hazard ratio (HR) for developing diabetes was 1.65 (95%CI, 1.07–2.54), with a significant linear trend between the self-reported frequency of difficulty initiating sleep and the risk of diabetes (p = 0.007). The positive associations with diabetes remained after adjusting for possible confounding factors. In model 1, the multivariate adjusted HR for diabetes was 1.39 (95%CI, 1.04–1.88) for participants with a moderate frequency of difficulty initiating sleep, and 1.63 (95%CI, 1.04–2.59) for a high frequency, with the linear trend remaining significant (p = 0.011). Adjusting for sleep duration (model 2) did not attenuate the hazard ratios. The effect of frequency of difficulty initiating sleep did not vary with gender (p = 0.82, 2 df, χ^2 ^= 0.91, model 2). In contrast to the difficulty in initiating sleep, a higher frequency of difficulty maintaining sleep was not significantly associated with risk of developing incident diabetes, even after adjusting for the confounding factors (Table 2).

**Figure 1:**
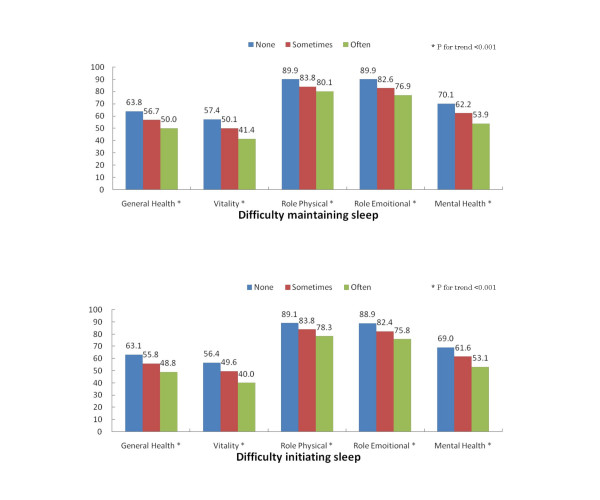
**Relation between difficulty initiating and maintaining sleep and SF36 domain scores**. Mean scores for the sub-domains of the SF-36 at baseline for different categories of difficulty initiating sleep and difficulty maintaining sleep (none; sometimes; often). SF-36 sub-domains include general health, vitality, role-physical, role-emotional, and mental health.

To assess the construct validity of the question evaluating the frequency of difficulty initiating and maintaining sleep, we first determined the association between these questions and sleep duration (Table 1). The more often that participants experienced difficulty initiating or maintaining sleep, the shorter sleep duration was reported. Of those who reported difficulty initiating sleep, the proportions whose sleep duration was less than 6 hours were respectively 14.3%, 16.9%, and 30.3% for none, sometimes, and often; a significant association was observed (p < 0.001). Similarly, for those who reported difficulty maintaining sleep, the proportions whose sleep duration was less than 6 hours were 14.1%, 15.9%, and 29.7% for none, sometimes, with a significant association (p < 0.001) (results not shown in Table). Next, we evaluate the association with SF-36 sub-domain scores. The reported frequency of difficulty initiating sleep was significantly associated with all five of the SF-36 domains measured at baseline (Figure 1). There was a statistically significant linear inverse association between the frequency of difficulty initiating sleep and the SF-36 scores (p for trend < 0.001 for all measures).

## Discussion

We found a modest but significant positive association between a high self-reported frequency of difficulty initiating sleep and a higher risk of developing diabetes, even after adjusting for large number of possible confounders in their association. This effect was not attenuated by BMI or duration of sleep. Contrary to previous reports [6-8], a shorter sleep duration and difficulty maintaining sleep was not associated with a higher risk of diabetes mellitus in healthy Asian workers.

Epidemiological studies have evaluated the association between sleep and the risk of developing diabetes mellitus. A short sleep duration was associated with a higher risk of developing diabetes, but association with longer sleep durations was not consistently found [6-8]. Mallon et al. reported that difficulty maintaining sleep, but not difficulty initiating sleep, was associated with the risk of diabetes. In contrast, Nilsson et al. found an association between those who had difficulty falling asleep or who regularly used hypnotics and the development of diabetes, in a cohort of 6599 middle-aged men in Sweden [25]. The present results nevertheless suggest that sleep duration is not associated with the risk of developing diabetes. Our study also differs from some other earlier studies. There are some possible explanations for this discrepancy. First, our study population is relatively lean (mean BMI, 22.6 kg/m^2^) compared to that studied by Ayas et al. (mean BMI, 24.6 kg/m^2^). As they pointed out, short sleep duration per se may not be a risk factor for diabetes; instead, a high BMI may worsen sleep quality. In fact, in their report, the relative risk of diabetes was no longer significant for short sleepers after adjustment for BMI; this may explain the discrepancy between the two studies. Second, most subjects in previous studies were not drawn from an Asian population, whereas all of the present study participants were Asian. The effect of short sleep time on the increased risk of diabetes may vary with ethnicity. Third, in our study, very few people had a sleep duration of less than 5 hours or more than 9 hours. This low variation of sleep duration limited our ability to detect any positive relation between short sleep duration and a higher risk of developing diabetes mellitus. Finally, the participants' normal sleep duration was self-reported. Self-reported sleep duration has a moderate degree of variation over time [26], and misclassification might bias the present findings toward the null.

Although the mechanism underlying the positive association between difficulty initiating sleep and diabetes is not clear, we are able to propose some candidates. First, some reports exist that sleep disorders are associated with increased activity of the sympathetic nervous system [27,28], which in turn is associated with increased glycogen breakdown and gluconeogenesis, and consequently induces insulin resistance. Second, it has been reported that psychological distress is associated with poor glycemic control [29-33]. In the present study, difficulty initiating sleep was significantly associated with a lower mental health sub-domain score in the SF-36 questionnaire, and this might be an intermediate mechanism in the association between quality of sleep and risk of diabetes.

The design of this study minimized the possibility that reporting of sleep duration or quality of sleep is biased by the diagnosis of diabetes, which could occur in case-control studies. Other strengths of our study include the long follow-up and the relative homogeneity of socioeconomic status and ethnicity among subjects. We also evaluated the construct validity of the sleep quality questionnaire, validating this tool for evaluating the association with the risk of diabetes.

We can suggest some possible limitations to this study. First, sleep duration and difficulty initiating sleep measurements were self-reported. Since the bias of the effect on the risk of diabetes should tend towards the null, our results for the association between difficulty initiating sleep and the risk of diabetes should stand, but there is room for improvement. Second, since this is an observational study, confounding factors could explain the findings; however, this possibility is limited by the design of the data collection and the adjustments made for the many factors that could mask the relation between sleep problems and incident diabetes. Because we used data from an intervention study including diet, physical activity, and cessation of smoking, this might be a confounder in the association between sleep and type 2 diabetes, but we believe the impact must be small because adjustment for intervention did not influence the results. Small sample size limited us to conduct sub-group analysis by gender, although we did not observe statistically significant interaction by gender. Finally, since our study population consisted of a relatively lean and young Asian population, caution should be used in extrapolating our results to more obese, to non-Asian, or to older groups.

## Conclusion

Results from the HIPOP-OHP suggest that difficulty initiating sleep, but not sleep duration or difficulty initiating sleep, is associated with a higher risk of diabetes in relatively healthy Asian workers, even after adjusting for a large number of possible confounders. These findings have implications for the prevention of diabetes.

## Competing interests

The author(s) declare that they have no competing interests.

## Authors' contributions

YH, SF, YS, TO, TT, and HU conceived the study and were involved in its design. YH performed statistical analysis. YH and YS drafted the initial manuscript for journal submission and participated in revisions. TO, TT, and HU coordinated the data collection. All authors read and approved the final manuscript.

## Appendix

### HIPOP-OHP Research group

Chairman: Hirotsugu Ueshima (Department of Health Science, Shiga University of Medical Science, Otsu, Shiga).

Participants: Akira Okayama (Department of Preventive Cardiology, National Cardiovascular Center, Osaka); Kiyomi Sakata and Keiko Tsuji (Department of Hygiene and Public Health, Iwate Medical University, School of Medicine, Iwate); Katsushi Yoshita (Department of National Nutrition Survey and Health Informatics, National Institute of Health and Nutrition); Toru Takebayashi and Yuriko Kikuchi (Department of Preventive Medicine and Public Health, School of Medicine, Keio University); Hideaki Nakagawa and Katsuyuki Miura (Department of Epidemiology and Public Health, Kanazawa Medical University); Hiroshi Yamato (Institute of Industrial Ecological Science, University of Occupational and Environmental Health); Nagako Chiba (Department of Human-Life, Tsukuba International Junior College); Masahiko Yanagita (Department of Nursing Science, Fukui Prefectural University); Kazunori Kodama, Fumiyoshi Kasagi and Nobuo Nishi (Department of Epidemiology, Radiation Effects Research Foundation), Yukinori Kusaka (Department of Environmental Health, Faculty of Medical Sciences, University of Fukui); Shigeyuki Saitoh (Second Department of Internal Medicine School of Medicine, Sapporo Medical University); Hideo Tanaka (Department of Cancer Control and Statistics, Osaka Medical Center for Cancer and Cardiovascular Diseases); Masakazu Nakamura (Cholesterol Reference Method Laboratory Network at Osaka Medical Center for Health Science and Promotion); Masakazu Nakamura and Yoshihiko Naito (Osaka Medical Center for Health Science and Promotion); Yasuyuki Nakamura (Cardiovascular Epidemiology, Faculty of Home Economics, Kyoto Women's University); Makoto Watanabe and Yoshikazu Nakamura (Department of Public Health, Jichi Medical School); Akira Babazono (Institute of Health Science, Kyushu University), Unai Tamura, Junko Minai, Zentaro Yamagata (Department of Health Sciences, School of Medicine, University of Yamanashi); Sumio Urano (Matsushita Health Care Center), Fujihisa Kinoshita (Wakayama Wellness Foundation); Isao Saitoh (Department of Public Health, Nara Medical University); Shinichi Tanihara (Department of Public Health, School of Medicine, Shimane University, Japan); Junko Tamaki (Department of Public Health, Kinki University School of Medicine); Osamu Tochikubo (Department of Public Health, Yokohama City University School of Medicine); Takeo Nakayama (Department of Medical System Informatics, Graduate School of Medicine and Faculty of Medicine, Kyoto University); Shunichi Fukuhara (Department of Epidemiology and HealthCare Research, Graduate School of Medicine and Faculty of Medicine Kyoto University), Yoshiharu Fujieda (Department of Health and Sport Sciences, Tokyo Gakugei University); Mariko Naito(Department of Preventive Medicine/Biostatistics and Medical Decision Making, Nagoya University Graduate School of Medicine); Shunsaku Mizushima (Department of Human Resources Development, National Institute of Public Health); Yuji Miyoshi (Tokyo central Clinic, Health Insurance Society of Meiji Yasuda Life Insurance Company); Takayo Tada (Department of Food Science, Faculty of Human Life Science, Mimasaka University); Taichiro Tanaka, Takashi Kadowaki, Toshimi Yoshida, Mami Ide and Tomonori Okamura (Department of Health Science, Shiga University of Medical Science, Otsu, Shiga).

## Pre-publication history

The pre-publication history for this paper can be accessed here:


